# Synthesis and Characterization of Innovative Double-Network Hydrogels with Potential as Adsorbent Materials for Wastewater Treatment

**DOI:** 10.3390/polym17040463

**Published:** 2025-02-10

**Authors:** Alexandra Vieru, Onur Yilmaz, Alina Gabriela Rusu, Cătălina Natalia Yilmaz, Alina Ghilan, Loredana Elena Nita

**Affiliations:** 1 Petru Poni Institute of Macromolecular Chemistry, Romanian Academy, 41A Grigore Ghica Voda Alley, 700487 Iasi, Romania; croitoriu.alexandra@icmpp.ro (A.V.); rusu.alinagabriela@yahoo.com (A.G.R.); alinush_dya@yahoo.com (A.G.); 2 Leather Engineering Department, Faculty of Engineering, Ege University, 35100 Izmir, Türkiye; onuryilma@gmail.com; 3 Biochemistry Division, Department of Chemistry, Faculty of Science, Dokuz Eylul University, 35210 Izmir, Türkiye; duncaty@gmail.com

**Keywords:** double network, tripeptide, anionic dye, homopolymer, supramolecular gel

## Abstract

Nowadays, large amounts of wastewater arise from various industrial applications. The discharge of wastewater into the environment represents a threat to the aquatic ecosystem and human health. Thus, in the present study, innovative double-network (DN) hydrogels with pH-sensitive features and applicability as adsorbents in the treatment of leather dye wastewater were prepared. The polyelectrolyte, poly(*N*,*N*-dimethylaminoethyl methacrylate (PDMAEMA), was obtained via the radical polymerization process, while the supramolecular structure was co-assembled through physical interactions. As a novelty, the double network was obtained through the interpenetration of the supramolecular network in the cross-linked polymeric one. The new hydrogels were physico-chemically and morphologically characterized by Fourier transform infrared spectroscopy (FTIR), scanning electron microscopy (SEM), and in terms of thermogravimetric analysis (TGA), swelling degree measurements, and dye adsorption studies. The DN hydrogels present interconnected macropores and high thermal stability. The swelling capacity of the dual network gels highlights a superadsorbent behavior at pH 3. Furthermore, the dye adsorption study highlights the effects of several variables (pH, concentration dose of adsorbent) on the ability of the gels to adsorb an anionic dye. The adsorption kinetics of the anionic dyes fitted the pseudo-first-order model (PFO). The estimated maximum adsorption capacities for the anionic dyes was 451 mg g^−1^ for PDMAEMA and 545 mg g^−1^ for DN gel.

## 1. Introduction

In recent decades, environmental concerns regarding water contamination have become increasingly significant [1]. According to the World Health Organization (WHO), billions of people suffer due to poor access to water. By 2050, it is predicted that water consumption will be much higher than today due to urbanization, population growth, and large-scale industrialization [1].

The leather industry, as a by-product processing sector of the meat industry, plays a significant role in the global economy, contributing over USD 200 billion annually across all related sectors. It is particularly vital for developing countries such as Brazil, China, India, Pakistan, Bangladesh, etc. as well as Europe, which accounts for 25% of global leather production, with major contributions from Italy, Spain, Portugal, Türkiye, etc. [2,3,4].

Leather processing involves extensive chemical reactions conducted in water, utilizing over 130 different chemicals. For every ton of raw hides/skins processed conventionally, approximately 450 kg of chemicals are used, of which only 72 kg are retained in the finished leather, while the remainder is discharged into 40–50 tons of wastewater [5,6]. This wastewater is characterized by high pH, a strong odor, and a dark brown color. Major pollutants include chromium, tannins, phenolic compounds, sulfides, and azo dyes, all of which are harmful to humans, animals, and plants [7,8,9]. The effluents generated during leather processing are challenging to treat using conventional methods due to the presence of dyes. Dyes, particularly azo dyes and metal complexes, pose significant environmental concerns as they can lead to acute and chronic toxicities [2,3].

Currently, numerous methods are involved in the treatment of wastewater from the leather industry. The traditional methods include filtration, sedimentation, chemical oxidation, and precipitation [9]. In addition to these, modern approaches such as adsorption, advanced oxidation processes, and electrocoagulation have been developed [10]. The adsorption process has gained considerable interest due to the selective retention of compounds on the solid surface of a substrate for wastewater treatment [11]. It is also considered a cost-effective and ecological technique for dye wastewater treatment presenting a high selective removal performance of acid and vat dyes, and also dispersive and reactive dyes [12]. By adjusting the properties of the aqueous phase, such as pH, both the ionization and surface charge of the adsorbent, as well as the adsorbate can be modified [13].

Therefore, numerous systems such as activated carbons, clays, carbon dots, polymeric membranes, hydrogels, or polymer brushes have been developed for the purification of water [14]. Hydrogels are considered the most cost-effective materials used for water purification. Literature data show that biopolymer-based adsorbents such as chitosan, agarose, cellulose, and starch have been used for removing dyes from wastewater [15]. Among them, systems based on stimuli-sensitive polymers have shown remarkable properties when subjected to various conditions, such as temperature-, pH-, light-, CO_2_- [16]. pH-sensitive polymers exhibit the ability to modify their inherent characteristics in response to pH variations. Polymers that have polar groups along the macromolecular chain behave as cationic polymers in acidic environments, and as anionic polymers in basic environments. Depending on their ionic composition, they exhibit different self-assembly behaviors. The changeover from the neutral state to the charged state facilitates the pH-sensitive polymers to absorb the oppositely charged ions and dyes due to the formation of electrostatic interactions. Due to their inherent characteristics, pH-sensitive polymers can be effectively used for wastewater treatment by forming interfacial bonding [17].

Hybrid DN hydrogels are an interesting alternative type of material used for sewage treatment [18]. The double-network structures show effectiveness in the adsorptive and regeneration process due to the synergistic effect of the multiple functionalities generated by both classes of compounds [19]. Thus, a DN hydrogel based on PDMAEMA, a pH-sensitive homopolymer, could automatically detect changes produced by the variation in pH and react to these changes by dye adsorption or desorption [20]. Alternatively, amino acids and short tripeptides can be used to customize DN gels in terms of hydrophilicity, surface properties, porosity, and density that enhance the adsorption process [21]. The lysine and gly-gly-gly blocked at the N-terminal with fluorenylmethoxycarbonyl protecting group (Fmoc) possess functional groups such as −NH−, −NH_2_, −OH, and − COO−, being able to enhance dye adsorption capacity [22]. Furthermore, while a tripeptide like Fmoc-Gly-Gly-Gly-OH might provide more functional hydroxyl groups that can be involved in the H bond formation between the DN hydrogel used as an adsorbent material and the dye, the modified amino acid Fmoc-Lys(Fmoc)-OH through its abundant active amino and carboxyl functional groups can imprint on the DN gel pH adaptability and reusability [23]. In the leather coloring process, pH is an essential factor because it ensures the quality of the final leather products. The pH of leather effluents was found to be in a wide range from 6 to 9 due to the use of various alkaline chemicals in leather processing [24,25]. Particularly, dyeing wastewater is highly acidic due to the multiple additions of acids (e.g., formic acid used as a dye-fixing agent [26]) during the dyeing steps [27]. Therefore, functional DN gels with sensitivity to different pH and weak reversible non-covalent bonds can significantly contribute to the treatment of leather-dyeing wastewater [28,29].

Consequently, the present study aimed to develop DN gels through the in situ interpenetration of two networks as a cost-effective solution for wastewater treatment resulting from the dyeing process of the leather. For DN gel preparation, the first network was formed by crosslinking PDMAEMA with *N,N*-methylene bis-acrylamide, while the lysine and gly-gly-gly blocked at the N-terminal with Fmoc were combined to form the second network. The two networks’ interpenetration offers the advantage of obtaining DN hydrogels that intrinsically exhibit changes in their morphology, mechanical strength, swelling ability, and diffusibility in response to pH variations. Therefore, the innovative DN gel obtained by combining two classes of compounds exhibits remarkable synergistic properties due to its chemical composition. The chemical versatility of this material is given by the ester and tertiary amino groups, attached to the side chain of the PDMAEMA homopolymer, but also the hydrophobic aromatic Fmoc and hydroxyl groups from the amino acid/tripeptide. Thus, the formation of non-covalent bonds, such as hydrogen bonds, electrostatic and hydrophobic interactions, and π–π interactions with various organic or inorganic moieties, increases the adsorption capacity of DN gels.

## 2. Materials and Methods

### 2.1. Materials

The monomer used to synthesize double-network gels was 2-(dimethylamino)ethyl methacrylate (DMAEMA), mol. formula: C_8_H_15_NO_2_ supplied by Alfa Aesar (Haverhill, MA, USA). Before use, DMAEMA was passed through an ion exchange column to remove the inhibitor. Ammonium persulfate (APS), mol. formula: (NH_4_)_2_S_2_O_8_) and *N*,*N*,*N*′,*N*′-tetramethyl ethylenediamine (TEMED), mol. formula: (CH_3_)_2_NCH_2_CH_2_N(CH_3_)_2_ were purchased from Merck (Burlington, MA, USA) and constituted the initiation system for PDMAEMA homopolymer synthesis. *N*,*N*′-methylene-bis-acrylamide (MBAAm) cross-linking agent, mol. formula: C_7_H_10_N_2_O_2_ was acquired from Merck. The following compounds were used to prepare the supramolecular structure: Fmoc-Lys(Fmoc)-OH amino acid, mol. formula: C_36_H_34_N_2_O_6_ acquired from Sigma-Aldrich (Darmstadt, Germany), and Fmoc-Gly-Gly-Gly-OH tripeptide, mol. formula: C_21_H_21_N_3_O_6_ supplied by Bachem (Bubendorf, Switzerland). Dimethyl sulfoxide (DMSO), and sodium hydroxide (NaOH) solvents were purchased from Fluka (Buchs, Switzerland) and Sigma-Aldrich, respectively. The phosphate buffer solution (PBS) with pH 7.4 and 0.01 M concentration was prepared according to the standard protocol. An anionic acid dye, Sellaset Blue HN (TFL Ledertechnik GmbH, Rheinfelden, Germany), mol. formula: (C_36_H_19_CrK_2_N_5_NaO_12_S_2_) used in the adsorption studies was donated by Kepler Deri Tek. San. ve Tic. A.Ş. (Istanbul, Turkiye).

### 2.2. Synthesis of the PDMAEMA Network

**Figure 1:**
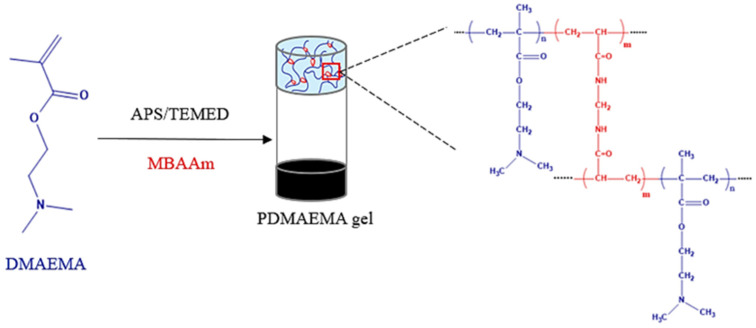
Schematic representation of the PDMAEMA-based synthetic network.

The synthetic network was prepared by using a redox initiation system (APS/TEMED) to polymerize the DMAEMA monomer by radical polymerization in the presence of water. The MBAAm was used as the cross-linker for the polymer chains. The volumetric ratio of 5% APS to 5% TEMED was 10:1. A schematic representation of the PDMAEMA network formation is shown in Figure 1.

### 2.3. Preparation of the Supramolecular Structure

**Figure 2:**
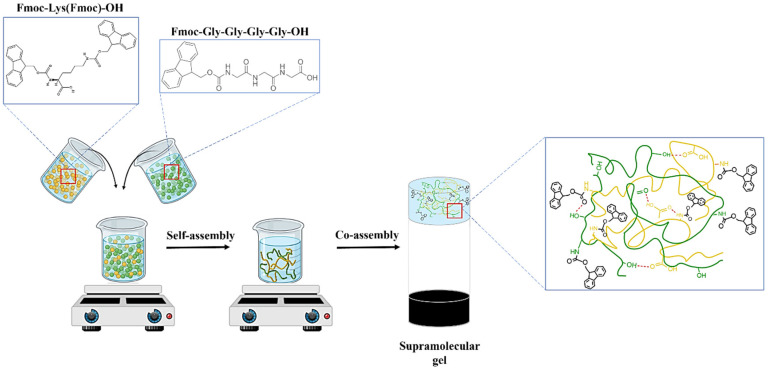
Schematic representation of the supramolecular network formation.

The preparation of the supramolecular network (S_1_) based on lysine and gly-gly-gly blocked at the N-terminal with Fmoc was carried out according to a previous study [29]. Briefly, the modified lysine (M_1_) and gly-gly-gly (M_2_) powders were dissolved in a polar solvent, DMSO, then 0.01 M PBS with pH 7.4 was slowly added. The DMSO/PBS ratio in the supramolecular gel was 1:61, while the co-partner solutions were co-assembled in 1:1 and 1:3 volumetric ratios, respectively. The formation of transparent supramolecular networks, based on physical interactions such as H bonds, π–π stacking, or electrostatic interactions, is presented in Figure 2.

### 2.4. Synthesis of DN Gels

**Figure 3:**
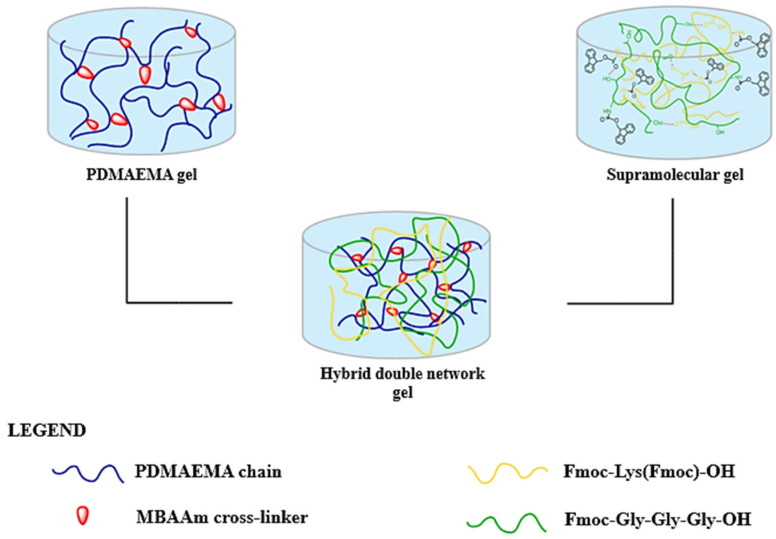
Schematic representation of the hybrid double-network formation.

The formation of DN hydrogels was achieved by immersing the S_1_ supramolecular network into the PDMAEMA-based synthetic network before it was fully structured, as shown in Figure 3. The method’s principle is based on the diffusion and interpenetration of the supramolecular network in the synthetic one. The samples were left at room temperature (25 °C) for 24 h, and the composition of the gels is detailed in Table 1.

The gels were lyophilized and then placed in dialysis membranes and purified. After purification, the gels were lyophilized again.

### 2.5. Characterization by Fourier Transform Infrared Spectroscopy

The structural characterization was conducted with a Vertex 70 spectrophotometer from Bruker, Bremen, Germany. The lyophilized gels were powdered and then mixed with potassium bromide (KBr). The mixture was then compressed at a pressure of 10 tons to create pellets. The recorded obtained FTIR spectra were processed using OPUS 6.5 software (Bruker).

### 2.6. Thermal Properties

The thermal properties of the PDMAEMA-based gels were analyzed using the STA 449 F1 Jupiter (Netzsch, Selb, Germany). Initially, the lyophilized gels weighing approx. 10 mg were placed in an open Al_2_O_3_ crucible. The thermal degradation occurred in the temperature range of 30–670 °C with a heating rate of 10 °C/min, and in an atmosphere of N_2_ with a purity of 99.99% and a gas flow rate of 40 mL/min. Data were collected and processed using Proteus^®^ 5.0.1 software (Visalia, CA, USA).

### 2.7. Morphological Properties

The gel structures’ morphology was analyzed by the Quanta 200 electron microscope (FEI Company, Hillsboro, OR, USA). The materials were frozen with liquid nitrogen, and placed on colloidal copper supports. The gold sputter-coated area was investigated in the high-vacuum mode using a 30 kV scanning electron microscope.

### 2.8. Swelling Degree

Each freeze-dried gel (PDMAEMA, and PDMAEMA_(S_1_ 1:3)) was weighed and immersed in PBS (pH 3, 4, and 5) at room temperature (25 °C) until swelling equilibrium was investigated. At regular intervals, the swollen samples were taken out from the media, the excess solution was blotted with filter paper, and then the samples were weighed. After each weighting, the samples were immersed back in the same media. All materials were analyzed in triplicate. The swelling degree was calculated using Equation (1) as follows:

(1) $$Swelling\;degree\;(\%)=(\frac{Ws-Wd}{Wd})\times 100$$

where:

Wd—weight of dry samples;
Ws—weight of wet samples.

### 2.9. Adsorption Study 

The adsorption capacities of PDMAEMA and PDMAEMA_(S_1_ 1:3) gels regarding the removal of anionic dyes were determined by performing batch adsorption experiments by varying different parameters (adsorbent dose, pH, time, dye concentration). The materials used in the adsorption study were used in a freeze-dried state. Table 2 presents the specific conditions used for the adsorption study.

Adsorption kinetics were determined by using 20 mg of freeze-dried adsorbent that was placed in flasks with 25 mL of Sellaset Blue HN dye solution of initial concentration (250 mg L^−1^). The flasks were gently shaken for 0–24 h at 25 °C, and at predetermined time intervals, the supernatant was collected. Subsequently, the amount of dye remaining in the supernatant was determined by measuring the absorbance at 580 nm using a Shimadzu spectrophotometer. The efficiency of anionic dye removal and adsorbed amount at time t and equilibrium were estimated by Equations (2) and (3).

(2) $$Dye\;removal\;efficiency\;(\%)=(\frac{C_{0}-Ce}{C_{0}})\times 100$$

where:

C_0_ represents the concentration of the stock anionic dye solution (mg L^−1^);
Ce represents the concentration of the dye solution at equilibrium (mg L^−1^).

(3) $$q_{e}=\frac{C_{0}-C_{e}}{M}\times V$$

where:

q_e_ represents the adsorption ability (mg g^−1^) of the adsorbent at equilibrium (mg g^−1^);
M represents the dose of adsorbent (mg);
V represents the volume of the dye solution (L).

The kinetic behavior of PDMAEMA and PDMAEMA_(S_1_ 1:3) gels toward the anionic dye was examined using the pseudo-first-order [30] and pseudo-second-order (PSO) [31] models. These kinetic models can be mathematically represented by Equations (4) and (5) as follows:

(4) $$\ln\left(q_{e}-q_{t}\right)=lnq_{e}-\frac{k_{1}}{2.303}t$$

and

(5) $$\frac{t}{q_{e}}=\frac{1}{k_{2}q_{e}^{2}}+\frac{1}{q_{e}}t$$

where:

q_e_ is the quantity of adsorbed dye at equilibrium (mg g^−1^);
q_t_ represents the quantity of adsorbed dye at specific intervals (mg g^−1^);
k_1_ represents the pseudo-first-order sorption rate constant (h^−1^);
k_2_ represents the pseudo-second-order sorption rate constant (g mg^−1^ h^−1^).

Furthermore, the diffusion mechanism was determined using the Weber–Morris [32] intraparticle diffusion (IPD) model, as follows:

(6) $$q_{t}=k_{p}t^{0.5}+C$$

where:

q_t_ represents the quantity of dye sorption at specific intervals (mg g^−1^);
k_p_ represents the intraparticle diffusion rate constant (mg g^−1^ h^−1/2^);
C represents the intercept.

### 2.10. Reusability Study

The reusability of the adsorbent materials was assessed by immersing them in methanol until the dye was removed. When the materials were clear, they were left at room temperature to dry and subsequently immersed again in the dye solution to perform the adsorption-desorption cycles.

### 2.11. Statistical Analysis

The data represent the average of three experiments and include the standard error of the mean (S.E.M.). To analyze the results, a one-way ANOVA with Tukey’s test was used to determine the statistical differences between data. Additionally, a one-way ANOVA with Tukey’s test and bivariate Pearson correlation were used to compare the samples resulting from the investigations.

## 3. Results

### 3.1. DN Gel Structural Characterization by Fourier Transform Infrared Spectroscopy

**Figure 4:**
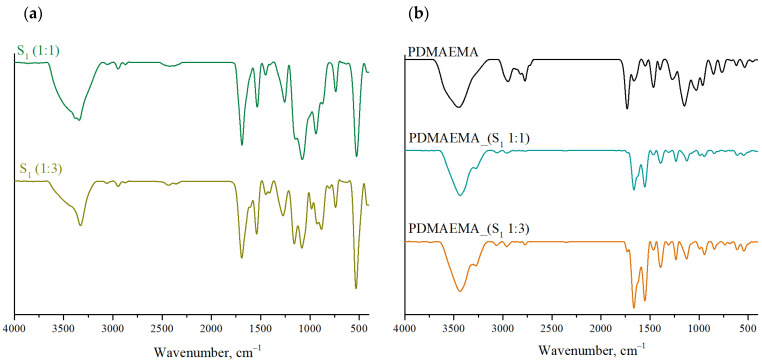
FTIR spectra of (**a**) the S_1_ supramolecular system co-assembled in 1:1 and 1:3 ratio, and (**b**) PDMAEMA and DN gels.

As can be seen in Figure 4b, the FTIR spectra of the DN systems present broad bands in the region of 3400 cm^−1^ and 3250 cm^−1^ characteristic for –OH and –NH groups in the structure of the co-partners M_1_ and M_2_ [33]. Specific bands for S1 supramolecular system (Figure 4a) are the type I amide band, due to the stretching vibrations of the C=O bond, which is found at approximately 1665 cm^−1^, followed by the type II amide band recorded at ~1553 cm^−1^, as a result of N–H bond bending vibrations and the type III amide band generated by C–N bond vibrations present in the 1235 cm^−1^ region. The characteristic bands for the Fmoc aromatic group are correlated with the stretching vibrations of the C–H bond, ~3064 cm^−1^, respectively, the vibrations of the C=C, C–C, or C–H groups found in the region 1000 cm^−1^–600 cm^−1^ [34].

The FTIR spectra of PDMAEMA, PDMAEMA_(S_1_ 1:1), and PDMAEMA_(S_1_ 1:3), shown in Figure 4b, reveal several characteristic peaks. For the PDMAEMA gel, the band at 3455 cm^−1^ corresponds to N–H stretching vibrations, characteristic for the acrylamide units. The absorption band at 2953 cm^−1^ is associated with C–H stretching vibrations from the methyl group, while the peaks at 2827 cm^−1^ and 2773 cm^−1^ appeared due to C–H stretching in the dimethylamino (–N(CH_3_)_2_) structure. Additionally, the band at 1736 cm^−1^ is characteristic of C=O stretching in ester groups, and the absorption band at 1468 cm^−1^ is correlated to methylene bending vibrations. Another band characteristic of the polymer appeared at 1154 cm^−1^ as a result of the stretching vibrations characteristic of the C–N group.

### 3.2. Thermal Stability Study

**Figure 5:**
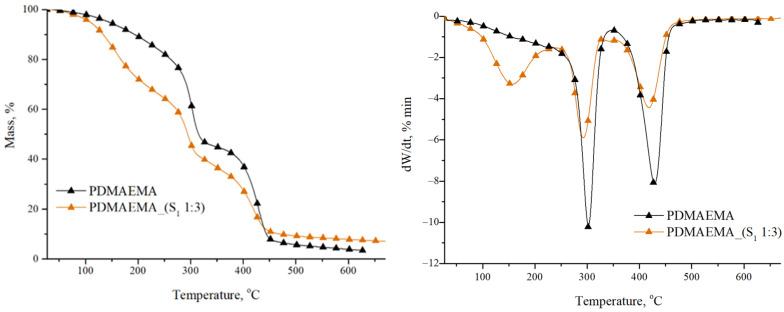
TGA graphs of the PDMAEMA network and PDMAEMA_(S_1_ 1:3) DN gel.

Figure 5 shows the thermal stability of the PDMAEMA homopolymeric network and PDMAEMA_(S_1_ 1:3) DN gel determined by thermogravimetric analysis.

The PDMAEMA network exhibited two main stages of decomposition. The first decomposition stage occurred within the 275–300 °C range having a significant mass loss of over 55 wt. %, as reported in the literature [35]. According to the study realized by Stawski et al. [36], in this stage, the oxidation and degradation of the dimethyl aminoethyl fragment (–CH_2_CH_2_N(CH_3_) from the side chain took place. The second stage of decomposition occurred at T_peak_ 425 °C. The mass loss of 41 wt. % is correlated with the carbonation process of the main polymer chain [37,38].

From Figure 5 it can be seen that the PDMAEMA_(S_1_ 1:3) DN gel shows three stages of decomposition as a result of the formation of numerous intermolecular H bonds between the S_1_ supramolecular system and PDMAEMA synthetic network. The first thermal decomposition stage with a T_peak_ at 156 °C and a weight loss of over 32 wt. % can be attributed to water and solvent residue evaporation, in accordance with the literature data [39,40]. The second stage presented a T_peak_ at 294 °C, and the 33 wt. % mass loss is correlated with the cleavage of hydroxyl, amine, and carboxyl groups from amino acid/tripeptide structure [41]. The breaking of the amide bonds before the (-CH_2_-)_4_ side chain occurred as a result of the lower C-N bond dissociation energy compared to the C-C bond energy [36]. The final stage of decomposition was attributed to the breaking of chemical bonds from the carbamate group in the Fmoc structure, as well as from the synthetic polymer PDMAEMA, which shows a mass loss of 26 wt. %.

Table 3 summarizes the main parameters of thermal decomposition for the synthesized gels. As can be seen, PDMAEMA_(S_1_ 1:3) has a higher value of residual mass (7.07%) than PDMAEMA (3.37%). This aspect can be correlated with the presence of the S_1_ supramolecular network in the DN gel. The multiple –CO–NH– bonds present in the structures of M_1_ and M_2_ precursors lead to the formation of an additional number of intra- and intermolecular bonds.

### 3.3. Morphological Study

**Figure 6:**
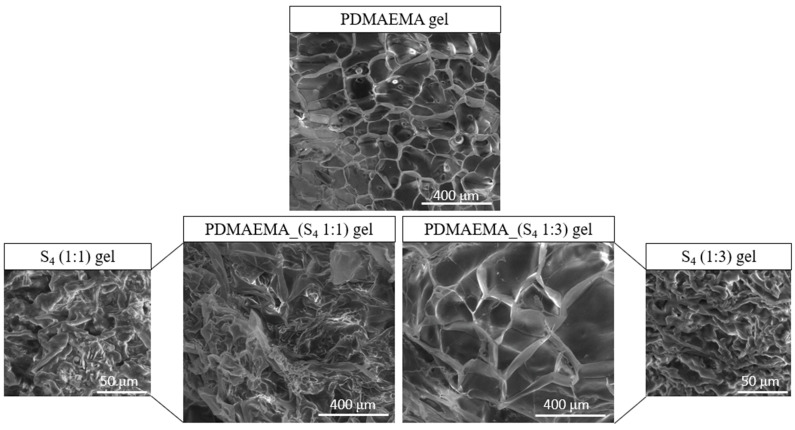
SEM images of the S_1_ supramolecular structure co-assembled in 1:1 and 1:3 ratios, PDMAEMA gel, and the DN gels.

The recorded SEM images of the S_1_ (1:1 and 1:3 ratio), PDMAEMA, and DN gels are presented in Figure 6. The precursor supramolecular networks obtained by the co-assembly of M_1_ and M_2_ show a fibrous morphology, the appearance of the fibers being given by the ratio between the two co-partners. The S_1_ co-assembled in a 1:1 ratio presents a dense network, while the presence of M_2_ in a higher ratio leads to an entangled fibrous network. The pristine PDMAEMA gel presents a homogeneous network, with a “honeycomb” appearance and uniformly distributed pores. The interaction between the supramolecular network (S_1_ co-assembled in a 1:1 ratio) and the PDMAEMA-based synthetic network leads to the appearance of a less ordered DN system as a result of the free Fmoc groups (which are not involved in π–π overlapping). In the case of PDMAEMA_(S_1_ 1:3), a network with interconnected pores, similar to that of the pristine PDMAEMA gel, was observed with slightly increased pore sizes.

**Figure 7:**
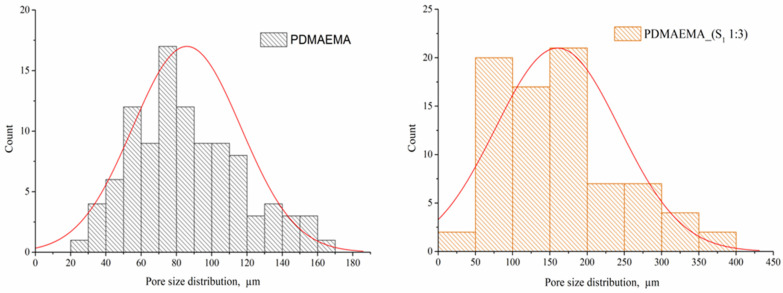
Pore size distribution histograms for PDMAEMA and PDMAEMA_(S1 1:3).

To determine the distribution of the pore size, only the PDMAEMA and PDMAEMA_(S_1_ 1:3) samples were selected, the others being excluded due to the lack of pores in the network. The size of pores was determined from SEM images using ImageJ 1.53t software (Bethesda, MD, USA). Figure 7 presents the corresponding histograms of the pore size distribution. The PDMAEMA network exhibited pores with dimensions between 20 μm and 160 μm. The presence of the S_1_ supramolecular system co-assembled in a ratio of 1:3 generated larger pores of up to 400 nm in the case of the PDMAEMA_(S_1_ 1:3) DN system. A unimodal Gaussian distribution can be observed for both systems [42,43].

### 3.4. Swelling Degree Behavior

**Figure 8:**
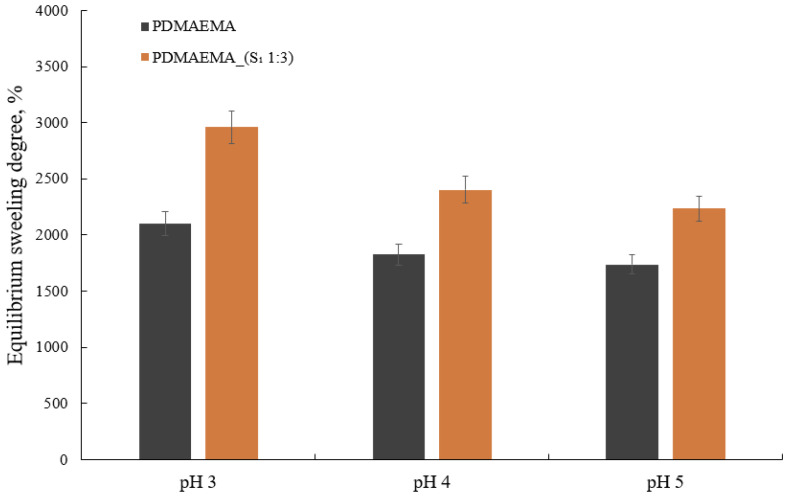
The equilibrium swelling degree of the PDMAEMA and PDMAEMA_(S_1_ 1:3) DN at different pHs.

The swelling kinetics of the double-network hydrogels are shown in Figure 8. Taking into account that the PDMAEMA-based network is a weak cationic polyelectrolyte with a pKa = 8.44 at 25 °C [44], the pH decreased below pKa generated the partial protonation of the tertiary amino groups [45]. Consequently, the electrostatic repulsion between the polymer chains caused them to drive away from each other and generated the “movement” of the tertiary amino groups, allowing the solvent to infiltrate the network’s meshes in large quantities.

Figure 8 summarizes the swelling degree at the equilibrium of PDMAEMA and PDMAEMA_(S_1_ 1:3) as a function of pH. The PDMAEMA network showed a higher swelling degree at pH 3 as a result of the protonation of the tertiary amino groups which generates an increase in the hydrophilicity of the polymer [37,46]. The PDMAEMA_(S_1_ 1:3) gel exhibited a higher swelling degree than PDMAEMA. This behavior can be attributed to the presence of the S_1_ co-assembled system and correlated with the synergistic effect offered by the chemical versatility of the amino acids and polyelectrolyte polymer. The capacity of both PDMAEMA and PDMAEMA_(S_1_ 1:3) hydrogels to swell decreased as the pH increased as a result of tertiary amino group deprotonation that implicitly determined the increase in hydrophobicity. Both hydrogels exhibited excellent swelling degrees ranging from ~2960% for PDMAEMA_(S_1_ 1:3) to ~2100% for PDMAEMA at 20 °C and pH = 3.

### 3.5. Dye Adsorption Study

The PDMAEMA_(S_1_ 1:3) gel was selected for the adsorption study due to its porous structure with uniformly distributed pores and a higher degree of swelling. The effectiveness of the PDMAEMA_(S_1_ 1:3) system as an efficient adsorbent for wastewater treatment was evaluated through a dye adsorption study.

The study was carried out using a dye solution at a concentration of 250 mg L^−1^ (except for the study where the concentration effect was determined) and an adsorbent dose of 0.02 g (except for the study on the effect of adsorbent dose where different adsorbent doses were used) as common parameters. PDMAEMA was used as a control.

#### 3.5.1. Effect of Adsorbent Dose

**Figure 9:**
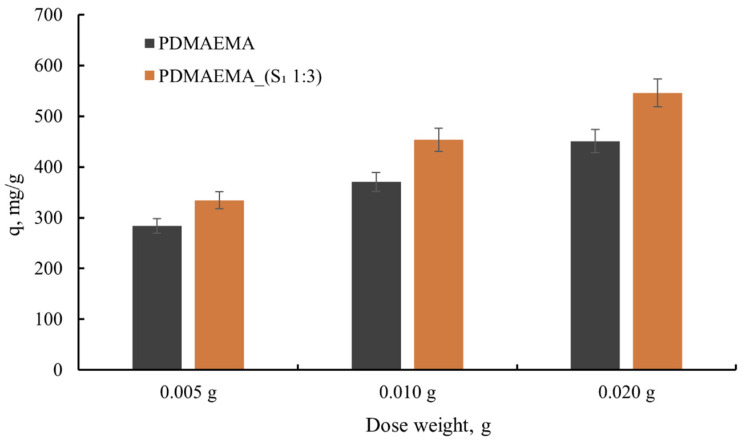
The dye adsorption capacity as a function of adsorbent dose.

Starting from the idea that adsorption takes place on the surface of the material [31], the adsorbent dose represents a constant that was studied to optimize efficient and sustainable dye wastewater treatment. Hence, a different dose of adsorbent (5, 10, and 20 mg) was involved in the wastewater treatment process, and the obtained results are presented in Figure 9.

As can be seen from Figure 9, the anionic dye uptake capacity of 5 mg of PDMAEMA_(S_1_ 1:3) was 334.1 mg g^−1^, while using a 10 mg adsorbent dose generated an increase in dye uptake to 453.6 mg g^−1^. In comparison, 20 mg of adsorbent dose presented a 545.6 mg g^−1^ dye uptake capacity. Taking into account that the PDMAEMA presents a lower adsorption capacity than PDMAEMA_(S_1_ 1:3), it can be established that the S_1_ supramolecular system contributes to the remarkable dye adsorption capacity of the DN gel. The high adsorption capacity of PDMAEMA_(S_1_ 1:3) can also be correlated with the presence of macropores (400 nm according to Figure 7) that allow for dye molecules to penetrate the network. The results indicate that by increasing the adsorbent dose from 0.005 mg to 0.02 mg, a larger surface area of the material is provided, and, implicitly, a larger number of adsorption sites. This aspect highlights the adsorption of several dye molecules [13].

#### 3.5.2. Effect of pH

Taking into consideration that the wastewater resulting from the leather dyeing steps has an acidic pH, the present study investigates the efficacy of the adsorbent material in removing an anionic dye from dye solutions with pH values between 3 and 5. Furthermore, pH is considered a critical factor responsible for the adsorption process for pH-sensitive materials [31].

**Figure 10:**
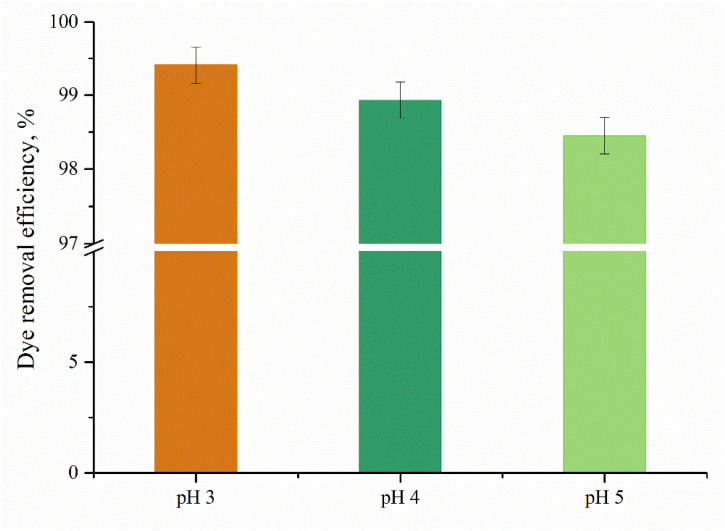
Adsorption capacity of PDMAEMA_(S_1_ 1:3) as a function of pH.

Figure 10 illustrates the way solution pH affects the anionic dye adsorption of PDMAEMA_(S_1_ 1:3). It could be observed that once the pH increases, the dye removal efficiency decreases slightly. An acidic pH value enables –NH_2_ to become NH_3_^+^ and to adsorb anionic dye molecules through electrostatic interactions. This electrostatic interaction is pH-dependent [47], with higher adsorption at lower pH values when more amino groups are protonated. PDMAEMA contains positively charged dimethylaminoethyl groups that can interact with anionic dyes through electrostatic attraction and the protonated amino groups on PDMAEMA provide a cationic interface that binds to the anionic dye molecules, forming an electrical double layer. Furthermore, PDMAEMA also exhibits hydrophobic properties due to the methyl groups, which can contribute to dye adsorption through hydrophobic interactions. The hydrophobic interactions created between the anionic dye molecules and the polymeric chains can lead to the formation of aggregates, enhancing the adsorption capacity. The hydrophobic interactions formed between anionic dye molecules and the PDMAEMA chains can promote the formation of aggregates, which enhances the adsorption capacity of the material.

#### 3.5.3. Effect of Contact Time

**Figure 11:**
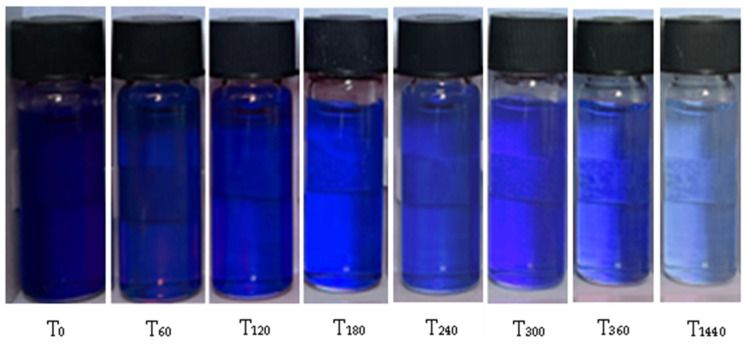
The appearance of dye solution over time as a function of the absorption ability of PDMAEMA_(S_1_ 1:3).

The PDMAEMA_(S_1_ 1:3) DN gel’s ability to adsorb anionic dye (250 mg L^−1^) was investigated in time (from 0 to 1440 min) at room temperatures (20 °C). Figure 11 presents the color change of the solutions in time.

The addition of 20 mg of PDMAEMA_(S_1_ 1:3) to the anionic dye solution led to gradual decoloration as the exposure time increased. After 24 h, the equilibrium condition was reached. The results revealed that the DN material initially exhibited a higher adsorption capacity. However, it was observed that as the contact time increased, the rate of adsorption decreased significantly, which can be attributed to the saturation effect. This indicates that once the adsorption sites become fully occupied, the material’s ability to adsorb additional dye molecules decreases, leading to a plateau in the adsorption process.

**Figure 12:**
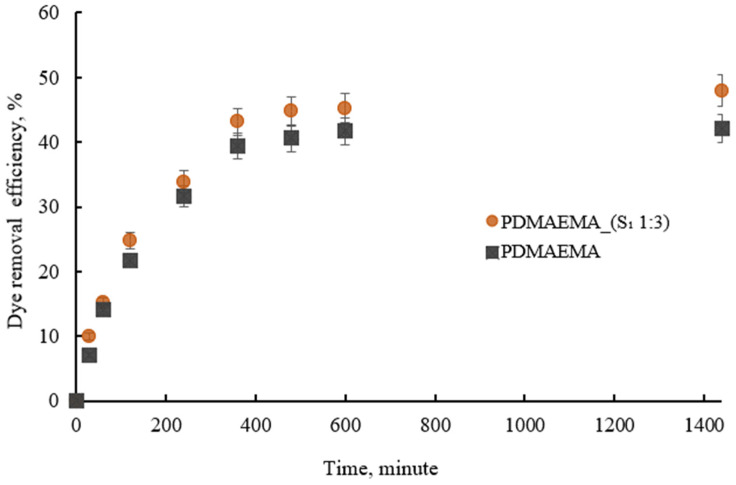
The dye removal efficiency of PDMAEMA and PDMAEMA_(S_1_ 1:3) in time.

Figure 12 shows that the PDMAEMA_(S_1_ 1:3) DN gel presented dye removal efficiency in time. Furthermore, the PDMAEMA_(S_1_ 1:3) gel outperformed the PDMAEMA gel in terms of adsorption efficiency due to the abundance of polar groups supplied by the supramolecular system in combination with the amine groups on the synthetic network. The amino acid and peptide residues can provide positively charged groups that interact with anionic dyes and the hydrophobic groups can participate in hydrophobic interactions with the dye molecules.

#### 3.5.4. Effect of Concentration

**Figure 13:**
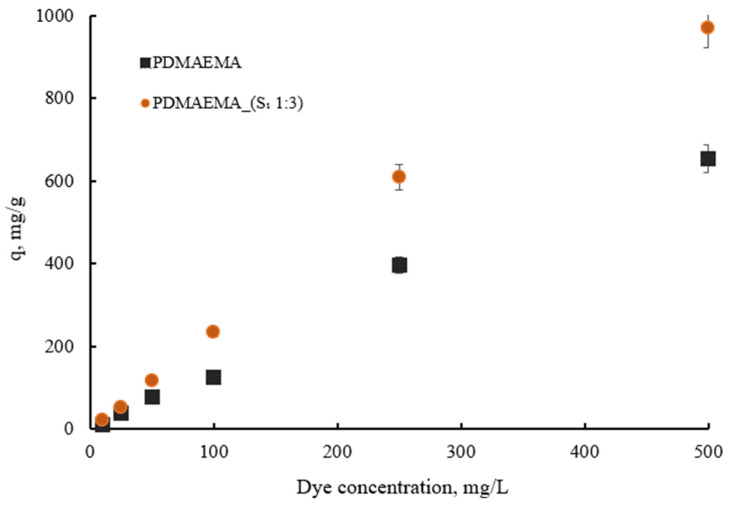
The dye adsorption capacity of PDMAEMA and PDMAEMA_(S_1_ 1:3) with variation in dye concentration.

Figure 13 depicts the dye adsorption capacity of PDMAEMA and PDMAEMA_(S_1_ 1:3) gels with varying initial SellaSet Blue HN dye from 20 mg L^−1^ to 500 mg L^−1^ concentrations. The results indicated that the maximum dye adsorption capacity for the synthetic network increased from 108 to 653.2 mg g^−1^, while for PDMAEMA_(S_1_ 1:3), it increased from 204 to 969.7 mg g^−1^. As observed, at higher dye concentrations, the absorption of the dye by the adsorbent was higher. This phenomenon is attributed to the intermolecular driving forces that develop between the adsorbent and the dye, which facilitate the movement of a larger quantity of dye molecules to the adsorbent’s surface, thereby occupying the available binding sites.

### 3.6. Regeneration Process

The study regarding material reusability highlights the PDMAEMA and PDMAEMA_(S_1_ 1:3) gel’s capacity in the removal of anionic dye after several cycles of adsorption/desorption. Following the adsorption cycle, the materials used as adsorbent were placed in methanol and subsequently dried at 20 °C. This method was cost-effective for the study and also provided feasibility for reusing the adsorbents [48,49].

**Figure 14:**
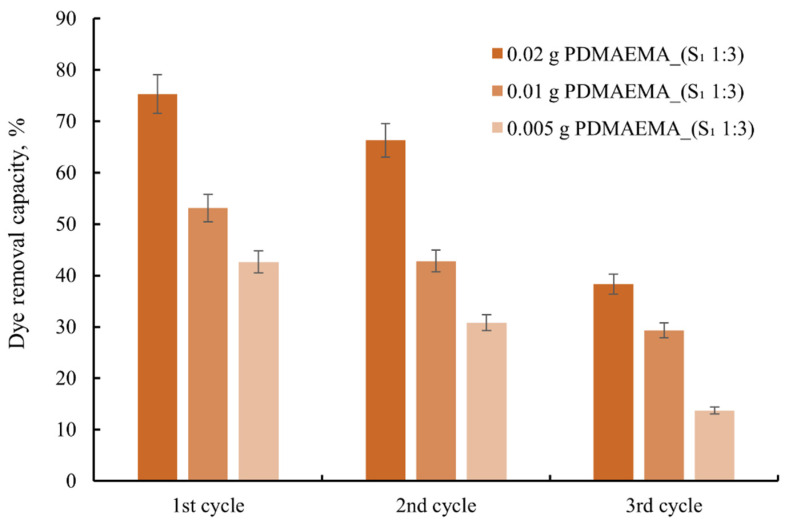
The reuse capacity of the studied materials for anionic dye.

As can be seen in Figure 14, in the first cycle PDMAEMA and PDMAEMA_(S_1_ 1:3) show a high absorption capacity of the dye compared to the dye solution, and in the following two cycles, the adsorption capacity decreases. This behavior is due to the shrinkage of the morphology of the material after the desorption process, or as a result of the dye molecules remaining in the material’s pores.

### 3.7. Kinetic Study of Anionic Dye Adsorption

**Figure 15:**
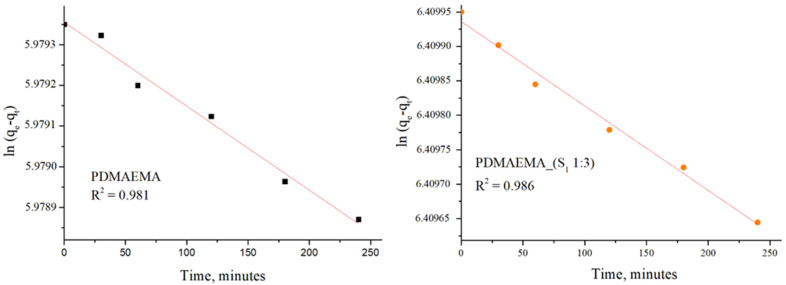
SellaSet Blue HN dye adsorption kinetic data fitted into the PFO model.

**Figure 16:**
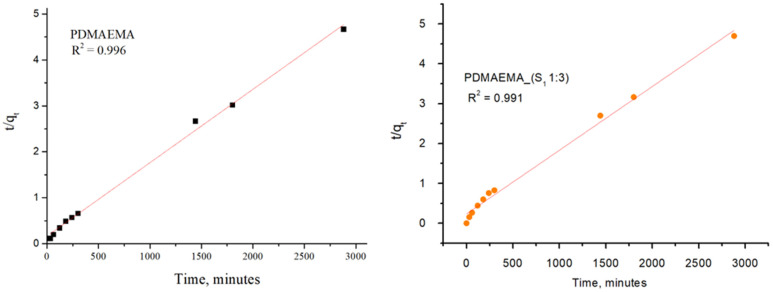
SellaSet Blue HN dye adsorption kinetic data fitted into the PSO model.

The adsorption kinetic behavior of the PDMAEMA and PDMAEMA_(S_1_ 1:3) gels toward anionic dye was examined by the PFO and PSO kinetic models. As depicted in Figure 15 and Figure 16, the results were fitted linearly to the two different kinetic models.

The PFO kinetic model forecasted for PDMAEMA_(S_1_ 1:3) q_e_ = 607.85 mg g^−1^ and R^2^ = 0.986, while the PDMAEMA presented q_e_ = 395.18 mg g^−1^ and R^2^ = 0.981. Furthermore, in the case of the PSO model, the values of q_e_ and R^2^ for the PDMAEMA_(S_1_ 1:3) were 632.91 mg g^−1^ and 0.994, while for the PDMAEMA gel were 625 mg g^−1^ and 0.996. However, the experimental adsorption capacity (q_exp_) for both PDMAEMA_(S_1_ 1:3) and PDMAEMA presents a better fit between the experimental results and the theoretical predictions provided by the PFO kinetic model.

The kinetic parameters were determined using the values of slopes and intercepts, which are presented in Table 4.

Therefore, the obtained results highlight that the adsorption process of PDMAEMA and PDMAEMA_(S_1_ 1:3) DN gel for the anionic dye could be more appropriately fitted to the PFO kinetic model. Even though the PFO kinetic model is well-fitted, some aspects limit and affect the kinetics of dye adsorption. These aspects remained unknown due to the dye adsorption mechanism on surfaces of a solid material typically involving multiple steps. To gain a complete understanding of the anionic dye adsorption mechanism onto PDMAEMA and PDMAEMA_(S_1_ 1:3), the intraparticle diffusion model was applied as follows.

**Figure 17:**
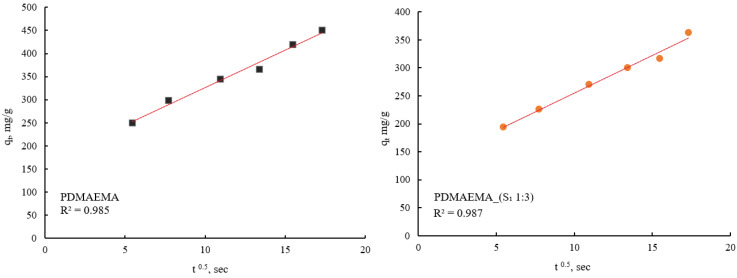
SellaSet Blue HN dye adsorption kinetic data fitted into the IPD.

From Figure 17 it can be observed that both PDMAEMA_(S_1_ 1:3) and PDMAEMA showed a single phase of adsorption, indicating that the dye molecules primarily are adsorbed onto the surface of the adsorbent material. This behavior suggests that the adsorption process is predominantly surface-controlled, rather than being influenced by diffusion into the bulk of the material.

Table 5 presents comparative data on removing dye molecules by PDMAEMA-based hydrogels to emphasize the ability of new PDMAEMA-based materials to adsorb anionic dye from wastewater.

Comparing the results obtained with data from the literature (Table 5), it can be seen that PDMAEMA_(S_1_ 1:3) DN gel showed good adsorption capacity for the SellaSet Blue HN anionic dye.

### 3.8. Adsorption Isothermal Study

Table 6 presents the parameters of the following isothermal adsorption models: Langmuir, Freundlich, Tempkin, and Dubinin–Radushkevich using linear equations.

According to the literature data, the Langmuir adsorption isotherm model describes monolayer adsorption on the surface of a homogeneous material with the same affinity of the binding sites for adsorption [54]. The Freundlich empirical model assumes that the adsorbent material has a heterogeneous surface, and the adsorption process is an interactive one [55], while the Temkin isotherm model is based on the principle that the adsorption process takes place through adsorbent–adsorbate interactions [56]. The last adsorption isotherm model included in the study is the Dubinin–Radushkevich model which describes that the adsorption is physical or chemical [56,57].

The obtained results show that the higher value of the correlation coefficient, R^2^ = 0.9434, for PDMAEMA was obtained in the case of the Freundlich model, while the adsorption process for PDMAEMA_(S_1_ 1:3) DN gel was described by Temkin isotherm model (R^2^ = 0.9820). Therefore, it can be concluded that the PDMAEMA network has a heterogeneous surface that allows for the interactive adsorption of dye molecules and the chemical versatility of PDMAEMA_(S_1_ 1:3) leads to adsorbent–adsorbate interactions.

## 4. Conclusions

The present study outlines the innovative DN gel synthesis based on PDMAEMA synthetic network and supramolecular co-assembled structure. The DN gel formation was carried out in three steps. First, the PDMAEMA network was obtained by in situ radical polymerization and chemical cross-linking in the presence of APS/TEMED as the redox system and BisAAm as the bcross-linker. In the second step, the co-assembled supramolecular structure was obtained by intermolecular physical interactions. In the last step, the interpenetration of the two precursor networks took place. Therefore, the formation of the DN gel was confirmed by FTIR analysis, while SEM microscopy of the PDMAEMA_(S_1_ 1:3) revealed the interconnected pore structure. The thermal behavior showed that the presence of the S_1_ supramolecular network in DN gel determines a higher thermal stability than PDMAEMA. Moreover, the DN gel was tested as a sorbent for an anionic dye by adsorption study as a function of pH change, adsorbent dose amount, dye concentration, and regeneration process. The experimental results demonstrated that the adsorption capacity of PDMAEMA and PDMAEMA_(S_1_ 1:3) was significantly influenced by the adsorbent dosage and the pH of the dye solution. The kinetics of SellaSet Blue HN anionic dye adsorption follow the PFO model. In summary, the absorption of anionic dyes by PDMAEMA and peptides is driven by electrostatic and hydrophobic interactions. Considering the value of dye removal efficiency and its environmentally friendly nature, it can be concluded that the PDMAEMA_(S_1_ 1:3) system is a suitable option as an absorbent material for leather dyeing wastewater treatment.

## Figures and Tables

**Table 1:** The composition and appearance of PDMAEMA_(S_1_ 1:1) and PDMAEMA_(S_1_ 1:3) double-network gels after vial test inversion.

Sample Appearance						
Control		DN gels				
PDMAEMA 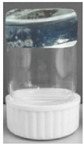		PDMAEMA_(S_1_ 1:1) 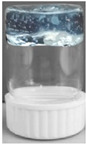			PDMAEMA_(S_1_ 1:3) 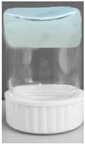	
Composition of 7 mL of gel						
Sample	DMAEMA Mmol	MBAAm mmol	APS mmol	TEMED mmol	M_1_ mmol	M_2_ mmol
PDMAEMA	2.9	0.32	0.021	0.066	-	-
PDMAEMA_(S_1_ 1:1)	2.9	0.32	0.021	0.066	0.033	0.048
PDMAEMA_(S_1_ 1:3)	2.9	0.32	0.021	0.066	0.016	0.072

**Table 2:** Conditions for the adsorption study.

Condition			
Adsorbent Dose mg	pH *	Time Minutes	Dye Concentration mg L^−1^
5	3	0–1440	1–500
10	4		
20	5		

* The pH was adjusted using either 0.1 M NaOH or HCl.

**Table 3:** Main parameters obtained from thermogravimetric analysis of PDMAEMA, and PDMAEMA_(S_1_ 1:3).

Control	Stage	T_onset_ (°C)	T_peak_ (°C)	T_20_ (°C)	T_50_ (°C)	W (%)	Residual Mass (%)
PDMAEMA	I	275	298	257	312	55.04	3.37
	II	387	425			41.59	
DN system	Stage	T_onset_ (°C)	T_peak_ (°C)	T_20_ (°C)	T_50_ (°C)	W (%)	Residual mass (%)
PDMAEMA_(S_1_ 1:3)	I	36.5	156	166	294	32.75	7.07
	II	268	294			33.69	
	III	366	418			26.49	

where T_onset_—the beginning of thermal decomposition; T_peak_—characteristic temperature of the maximum degradation rate; T_20_, T_50_—temperatures characteristic of weight losses of 20 and 50%; W—loss of weight.

**Table 4:** Kinetic parameters for SellaSet Blue HN anionic dye adsorption onto the PDMAEMA and PDMAEMA_(S_1_ 1:3) gels.

Calculated Values of Different Adsorption Kinetic Models				
Adsorption Kinetics Model	Linear Form	Constant	Calculated Value	
			PDMAEMA	PDMAEMA_(S_1_ 1:3)
Pseudo-first order	$\log\left(q_{e}-q_{t}\right)={\log q}_{e}-\frac{K_{1}}{2.303}t$	q_e_	395.18	607.85
		K_1_	−1.35 × 10^−9^	−6.52 × 10^−10^
		R^2^	0.981	0.986
		SD	0.000387	0.000303
Pseudo-second order	$\frac{t}{q_{e}}=\frac{1}{k_{2}q_{e}^{2}}+\frac{1}{q_{e}}t$	q_e_	625	632.91
		K_2_	0.000402	0.000241
		R^2^	0.996	0.994
		SD	1.515504	1.508638
Intraparticle diffusivity	$q_{t}=k_{p}t^{0.5}+C$	C	163.44	120.72
		K_diff_	16.237	13.41
		R^2^	0.996	0.994

**Table 5:** The comparison regarding PDMAEMA-based materials used as dye adsorbents and adsorption capacity.

PDMAEMA-Based Material	Dye Molecule	*Q*_m_ (mg g^−1^)	Reference
Karaya gum-*graft*-poly(2-(dimethylamino)ethyl methacrylate) gel	methylene blue	89.28	[50]
	indigo carmine	101.42	
GO-PDMAEMA nanocomposite	orange G	609.8	[51]
PEI/PDMAEMA gel	amaranth	757	[20]
	sunset yellow	744	
Carboxymethyl cellulose-g-poly(2-(dimethylamino) ethyl methacrylate) hydrogel	methyl orange	1825	[52]
Gellan gum-graft-poly(DMAEMA) hydrogel	methyl orange	25.8	[53]

**Table 6:** The calculated parameters of the isothermal adsorption models for PDMAEMA- and PDMAEMA_(S_1_ 1:3)-adsorbent materials.

Calculated Values of Different Adsorption Isotherm Models				
Adsorption Isotherm Model	Linear Form	Constant	Calculated Value	
			PDMAEMA	PDMAEMA_(S_1_ 1:3)
Langmuir	$\frac{C_{e}}{q_{e}}=\frac{1}{{K_{L}q}_{max}}+\frac{C_{e}}{q_{max}}$	q_max_	−158.73	−119.18
		K_L_	−0.012	−0.085
		R_L_	2.67	−0.303
		R^2^	0.8942	0.8702
Freundlich	$\ln q_{e}=\frac{1}{n}lnC_{e}+lnK_{F}$	n	1.048	2.401
		1/n	0.953	0.416
		K_F_	2.594	3.73
		R^2^	0.9434	0.9015
Temkin	$q_{e}={Blnk}_{T}+{BlnC}_{e}$	B_T_	166.81	243.70
		K_T_	14.71	10.06
		R^2^	0.8522	0.9820
Dubinin–Radushkevich	${ln\;q}_{e}={lnq}_{m}-\beta \varepsilon^{2}$	q_m_	403.42	148.41
		β	136.45	688.87
		E	0.0605	0.0269
		R^2^	0.3161	0.4072

## Data Availability

The data presented in this study are available in this article.

## Abbreviations

The following abbreviations are used in this manuscript:

DMAEMA	2-(dimethylamino)ethyl methacrylate
PDMAEMA	poly(*N*,*N*-dimethylaminoethyl methacrylate)
FTIR	Fourier transform infrared spectroscopy
SEM	Scanning electron microscopy
TGA	Thermogravimetric analysis
Fmoc	Fluorenyl methoxycarbonyl protecting group
S_1_	Supramolecular system based on Fmoc-Lys(Fmoc)-OH and Fmoc-Gly-Gly-Gly-OH
M_1_	Fmoc-Lys(Fmoc)-OH
M_2_	Fmoc-Gly-Gly-Gly-OH
DMSO	Dimethyl sulfoxide
DN	Double network
NaOH	Sodium hydroxide
PBS	Phosphate buffer solution
APS	Ammonium persulfate
TEMED	*N*,*N*,*N*′,*N*′-tetramethyl ethylenediamine
MBAAm	*N*,*N*′-methylene-bis-acrylamide
PFO	Pseudo-first-order model
PSO	Pseudo-second-order model
IPD	Intraparticle diffusion model
